# Lithium Enhances the GABAergic Synaptic Activities on the Hypothalamic Preoptic Area (hPOA) Neurons

**DOI:** 10.3390/ijms22083908

**Published:** 2021-04-09

**Authors:** Santosh Rijal, Seon Hui Jang, Soo Joung Park, Seong Kyu Han

**Affiliations:** Department of Oral Physiology, School of Dentistry & Institute of Oral Bioscience, Jeonbuk National University, Jeonju 54896, Korea; santoshrijal047@gmail.com (S.R.); sunnyjang@jbnu.ac.kr (S.H.J.); soopark@jbnu.ac.kr (S.J.P.)

**Keywords:** lithium, hypothalamic preoptic area neurons, GABAergic neurotransmission, patch-clamp, neuroendocrine axis

## Abstract

Lithium (Li^+^) salt is widely used as a therapeutic agent for treating neurological and psychiatric disorders. Despite its therapeutic effects on neurological and psychiatric disorders, it can also disturb the neuroendocrine axis in patients under lithium therapy. The hypothalamic area contains GABAergic and glutamatergic neurons and their receptors, which regulate various hypothalamic functions such as the release of neurohormones, control circadian activities. At the neuronal level, several neurotransmitter systems are modulated by lithium exposure. However, the effect of Li^+^ on hypothalamic neuron excitability and the precise action mechanism involved in such an effect have not been fully understood yet. Therefore, Li^+^ action on hypothalamic neurons was investigated using a whole-cell patch-clamp technique. In hypothalamic neurons, Li^+^ increased the GABAergic synaptic activities via action potential independent presynaptic mechanisms. Next, concentration-dependent replacement of Na^+^ by Li^+^ in artificial cerebrospinal fluid increased frequencies of GABAergic miniature inhibitory postsynaptic currents without altering their amplitudes. Li^+^ perfusion induced inward currents in the majority of hypothalamic neurons independent of amino-acids receptor activation. These results suggests that Li^+^ treatment can directly affect the hypothalamic region of the brain and regulate the release of various neurohormones involved in synchronizing the neuroendocrine axis.

## 1. Introduction

Gamma-aminobutyric acid (GABA) is well known to be a major inhibitory neurotransmitter in the central nervous system (CNS) [1]. Excitation and inhibition of neuronal activities in the CNS are balanced by GABAergic transmission, whose impairment can result in various CNS disorders [2,3]. Various physiological and pathological conditions continuously modulate the strength and polarity of GABAergic transmission in the CNS [4]. The hypothalamic area of the CNS contains GABAergic and glutamatergic neurons and their receptors that regulate various hypothalamic functions such as the release of neurohormones, control circadian activities [5]. Additionally, some hypothalamus areas receive GABAergic and glycinergic innervations from other brain areas [6,7,8].

In the hypothalamic preoptic area (hPOA), GABA and glutamate mediate most of the fast postsynaptic potentials/events, indicating that neuronal communications in the hypothalamic area are due to amino acid neurotransmitters [9]. GABA in the hypothalamic area exerts multiple effects on the hypothalamic-pituitary system. It is involved in the physiological control of anterior pituitary hormones [10], and regulation of the neuroendocrine system [6]. The hPOA is critically involved in several homeostatic processes such as sleep, reproduction, osmolality, body temperature, and behavior process as most brain regions have interconnections to the hPOA [11].

Lithium-ion (Li^+^) is known to exhibit a therapeutic effect in the treatment of some neurological disorders such as Parkinson’s diseases, Alzheimer’s diseases, and bipolar disorder [12,13]. Neurotransmitters such as GABA, glutamate, dopamine, glycine, and acetylcholine are modulated by lithium [13]. It has been reported that Li^+^ suppresses dopamine and glutamate transmissions but increases GABA neurotransmission at the neuronal level [14]. For example, granule cells (GCs) in the hippocampal dentate gyrus show increased GABAergic synaptic inputs to GCs by lithium [15]. Besides, Li^+^ can act on the second-messenger system at the intracellular and molecular level, thus, regulating neurotransmission [14].

Both in vivo and in vitro studies have suggested that Li^+^ can alter the release of several hormones such as prolactin, growth hormone [16], corticotropin-releasing hormone [17], arginine vasopressin [18], and opioid peptides like β-endorphin, dynorphin, and met-enkephalin from the hypothalamus [19]. Furthermore, Li^+^ can suppress the secretion of gonadotropins and gonadal hormones [20], decrease testosterone levels in male rats, and increase estrogen levels in female rats [21]. Several clinical cases have reported that Li^+^ can impact hypothalamic-pituitary-adrenal (HPA) [22,23], hypothalamic-pituitary-thyroid (HPT) [24], and hypothalamic-pituitary-gonadal (HPG) [25] axes. However, the effect of Li^+^ on hypothalamic neuronal excitability and the precise action mechanism involved in such an effect have not been fully understood yet. Therefore, the objective of this study was to investigate Li^+^ action on hypothalamic neurons using the whole-cell patch-clamp technique.

## 2. Results

### 2.1. Li^+^ Enhances the Frequency of Spontaneous Inhibitory Postsynaptic Currents (sIPSCs)

**Figure 1:**
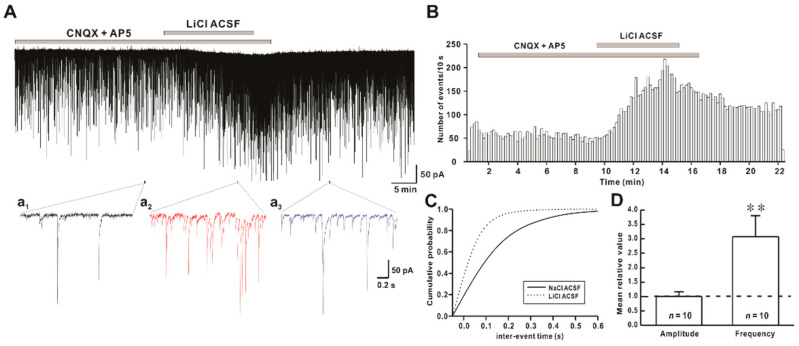
Effect of Li^+^ (126 mM) on spontaneous inhibitory postsynaptic currents (sIPSCs) on hypothalamic preoptic area (hPOA) neurons. (**A**) A representative current trace of sIPSCs recorded in the presence of Na^+^ and Li^+^ artifical cerebrospinal fluid (ACSF) on hPOA neurons at a holding potential of −60 mV. (**a_1_**–**a_3_**), sections of the current trace in Figure 1A show sIPSCs before, during, and after perfusion of Li^+^ at 2 s intervals, respectively. (**B**) A spike frequency histogram (bin size 10 s) of current traces in Figure 1A. (**C**) A cumulative probability plot of sIPSCs inter-event interval (IEI) in the presence of Na^+^ (solid line) and Li^+^ (dotted line). Note that the cumulative probability curve was left-shifted by Li^+^_,_ indicating the increase of sIPSCs frequency (Kolmogorov-Smirnov test, *p* < 0.05). (**D**) Mean relative amplitude and frequency of sIPSCs in LiCl ACSF compared to NaCl ACSF (** *p* < 0.01 by a paired *t*-test).

sIPSCs were recorded from hPOA neurons in the presence of ionotropic glutamate receptor blockers CNQX and AP5. Mean frequency and amplitude of sIPSCs under normal artifical cerebrospinal fluid (ACSF) conditions were 3.02 ± 1.04 Hz (*n* = 10) and 80.5 ± 12.6 pA (*n* = 10), respectively. In the same neuron, perfusion of LiCl ACSF rapidly and reversibly increased the frequency of sIPSCs. Mean frequency and amplitude of sIPSCs in hPOA neurons during Li^+^ ACSF exposure were 7.05 ± 1.91 Hz (*n* = 10) and 73.9 ± 12.1 pA (*n* = 10), respectively (Figure 1A). The time-frequency histogram showed an immediate and reversible increase in the frequency of sIPSCs by Li^+^ (Figure 1B). The cumulative probability curve (Figure 1C) showed a progressive leftward shift in the inter-event interval (IEI) of sIPSCs events shown in Figure 1A. The frequency of sIPSCs was significantly increased by Li^+^ application (relative frequency compared to control: 3.08 ± 0.72, *n* = 10, *p* < 0.01). However, the amplitude of sPSCs was not affected by Li^+^ (relative amplitude: 1.01 ± 0.17, *n* = 10, *p* > 0.05) (Figure 1D).

### 2.2. Li^+^ Enhances the Frequency of Miniature Inhibitory Postsynaptic Currents (mIPSCs)

**Figure 2:**
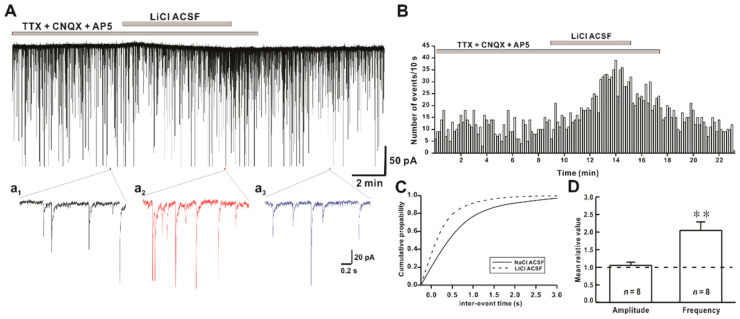
Effect of Li^+^ on miniature inhibitory postsynaptic currents (mIPSCs) on hPOA neurons. (**A**) A representative current trace of mIPSCs recorded in the presence of Na^+^ and Li^+^ ACSF on hPOA neurons at a holding potential of −60 mV. (**a_1_**–**a_3_**), sections of the current trace in Figure 2A show mIPSCs before, during, and after perfusion of Li^+^ at 2-s intervals, respectively. (**B**) A spike frequency histogram of the current trace in Figure 2A. (**C**) A cumulative probability plot of mIPSCs inter-event interval (IEI) in the presence of Na^+^ (solid line) and Li^+^ (dotted line). Note that the cumulative probability curve was left-shifted by Li^+^, indicating the increase of mIPSCs frequency (Kolmogorov-Smirnov test, *p* < 0.05). (**D**) Mean relative amplitude and frequency of mIPSCs in LiCl ACSF compared to NaCl ACSF (** *p* < 0.01 by paired *t*-test).

To determine if Li^+^ ion could affect action potential-independent release of an inhibitory neurotransmitter from presynaptic axon terminal, mIPSCs were recorded in the presence of tetrodotoxin (TTX, a voltage-gated Na^+^ channel blocker) and ionotropic glutamate receptor blockers CNQX and AP5. The frequency of mIPSCs was increased by Li^+^ ACSF application in all tested neurons, as shown in Figure 2A. Mean frequency and amplitude of mIPSCs under a normal ACSF condition were 1.94 ± 0.42 Hz (*n* = 8) and 57.1 ± 10.5 pA (*n* = 8), respectively. In the presence of Li^+^ ACSF, mean frequency and amplitude of mIPSCs in hPOA neurons were 4.44 ± 1.29 Hz (*n* = 8) and 57.2 ± 9.59 pA (*n* = 8), respectively. Time-frequency histogram of mIPSCs showed an increase in the frequency on Li^+^ exposure (Figure 2B). Li^+^ significantly shifted the cumulative frequency curve to the left (Figure 2C). The frequency of mIPSCs was significantly increased by Li^+^ application (relative frequency compared to control: 2.04 ± 0.24, *n* = 8, *p* < 0.01). However, the amplitude of sPSCs was not affected by Li^+^ (relative amplitude: 1.05 ± 0.09, *n* = 8, *p* > 0.05) (Figure 2D).

### 2.3. Li^+^ Increases the Frequency of GABAergic mIPSCs on hPOA Neurons

**Figure 3:**
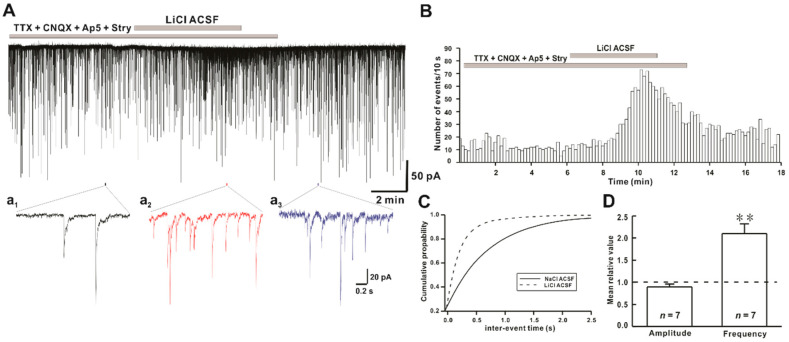
Effect of Li^+^ on GABAergic mIPSCs on hPOA neurons. (**A**) A representative current trace of GABAergic mIPSCs recorded in the presence of Na^+^ and Li^+^ ACSF on hPOA neuron. (**a_1_**–**a_3_**), sections of the current trace in Figure 3A show GABAergic mIPSCs before, during, and after perfusion of Li^+^ at 2-s intervals, respectively. (**B**) A spike frequency histogram of the trace in Figure 3A. (**C**) A cumulative probability plot of GABAergic mIPSCs inter-event interval (IEI) in the presence of Na^+^ (solid line) and Li^+^ (dotted line). Note that the cumulative probability curve was left-shifted by Li^+^, indicating the increase of GABAergic mIPSCs frequency (Kolmogorov-Smirnov test, *p* < 0.05). (**D**) Mean relative amplitude and frequency of GABAergic mIPSCs in LiCl ACSF compared to NaCl ACSF (** *p* < 0.01 by paired *t*-test).

In the CNS, GABAergic and glycinergic neurotransmissions are the major inhibitory inputs in neuronal regulation. The pre-optic area contains GABAergic neurons [26] and receives glycinergic intervention from a different region of the brain [7,8]. To determine whether GABA release might be involved in the increase of mIPSCs frequency by Li^+^, mIPSCs were recorded in the presence of strychnine, a glycine receptor antagonist. Mean frequency and amplitude of GABAergic mIPSCs under normal ACSF condition were 1.90 ± 0.41 Hz (*n* = 7) and 44.7 ± 7.7 pA (*n* = 7), respectively. The mean frequency of GABAergic mIPSCs in the presence of strychnine was increased to 3.65 ± 0.61 Hz (*n* = 7) by Li^+^. However, the mean amplitude was not changed (38.1 ± 5.03 pA, *n* = 7), (Figure 3A). The time-frequency histogram showed an immediate and reversible increase in the event of GABAergic mIPSCs in the presence of Li^+^ ACSF (Figure 3B). In the cumulative probability plot, the IEI of the GABAergic mIPSCs was shifted to the left, as shown in Figure 3A, indicating an increase in the frequency of GABAergic mIPSCs (Figure 3C). The frequency of GABAergic mIPSCs was significantly increased by Li^+^ application (relative frequency compared to control: 2.1 ± 0.22, *n* = 7, *p* < 0.01). However, the amplitude of sPSCs was not affected by Li^+^ (relative amplitude: 0.9 ± 0.06, *n* = 7, *p* > 0.05) (Figure 3D).

**Figure 4:**
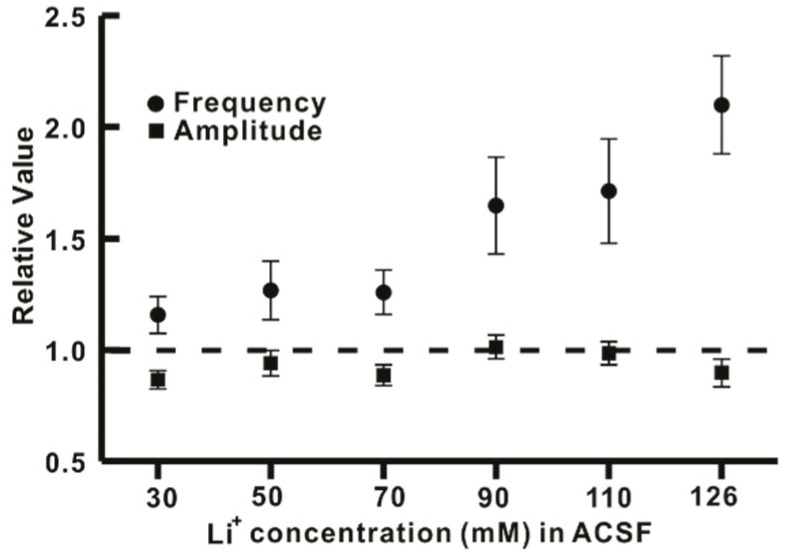
Concentration-dependent effect of Li^+^ on GABAergic mIPSCs. The graph shows a concentration-dependent increase in GABAergic mIPSCs frequency by Li^+^ without affecting the amplitude.

Furthermore, the concentration-dependent replacement of Na^+^ ions with Li^+^ ions in the ACSF solution augmented the frequency of GABAergic mIPSCs in a concentration-dependent manner without affecting the amplitude, as shown in Figure 4.

### 2.4. Li^+^ Perfusion Induces Repeatable Inward Currents on hPOA Neurons

**Figure 5:**
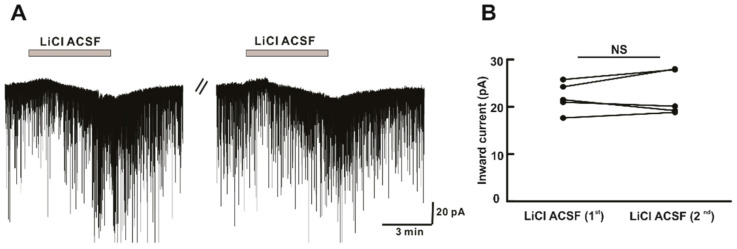
Li^+^ induces repeatable inward currents on hPOA neurons. (**A**) A representative current trace showing repeatable inward currents on hPOA neuron induced by perfusion of LiCl ACSF. (**B**) Before and after plot shows no significant difference in the mean inward currents between the first and the second perfusion of LiCl ACSF (*n* = 5, *p* > 0.05 by paired *t*-test). NS, not significant.

In addition to the effect of Li^+^ in postsynaptic currents, we observed a change in holding current on Li^+^ perfusion. In the whole-cell voltage-clamp experiment, a reversible inward current of magnitude (−21.6 ± 4.6 pA, *n* = 14) was observed in 14 of 18 neurons tested during Li^+^ ACSF perfusion. With successive application of LiCl ACSF, there was no significant difference in inward current induced during the first and second applications (Figure 5A). The mean amplitude of inward current (−22.0 ± 1.40 pA, *n* = 5) induced by the first application of LiCl ACSF was similar to that induced by the second application (−22.8 ± 2.1 pA, *n* = 5, *p* > 0.05) (Figure 5B). This indicated that Li^+^ induced a repeatable and non-desensitized response on hPOA neurons during the successive perfusion.

### 2.5. Li^+^ Directly Acts on hPOA Neurons and Its Action Is Independent of Amino-Acid Receptors

**Figure 6:**
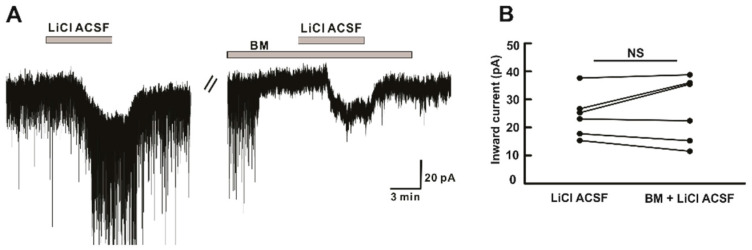
Li^+^ acts on postsynaptic hPOA neurons. (**A**) A representative current trace showing inward currents induced upon perfusion of LiCl ACSF. Li^+^ induced inward current persisted in the presence of BM (blocking mixture), including TTX (Na^+^ channel blocker), picrotoxin (GABA_A_ receptor blocker), strychnine (glycine receptor blocker), CNQX (non-NMDA glutamate receptor antagonist), and AP5 (NMDA receptor antagonist). (**B**) The before and after plot shows no significant difference in the mean inward current between Li^+^ alone and Li^+^ in the presence of blocking mixture (*n* = 6, *p* > 0.05 by the paired *t*-test). NS, not significant.

Next, we attempted to explore the direct action of Li^+^ ions on hPOA neurons. Neurons in the preoptic area can generate currents due to activation of GABA_A_, glycine, or ionotropic glutamate receptors [27,28]. Therefore, inward current induced by Li^+^ was recorded in the presence of blocking mixture (BM) including TTX (0.5 μM), picrotoxin (GABA_A_ receptor antagonist, 50 μM), CNQX (non-NMDA glutamate receptor antagonist, 10 μM), AP5 (NMDA glutamate receptor antagonist, 20 μM), and strychnine (glycine receptor antagonist, 2 μM). In the presence of BM, the inward current induced by LiCl ACSF was maintained (Figure 6A). There was a similarity in the mean amplitude of inward current induced by Li^+^ alone (−24.3 ± 3.21 pA, *n* = 6) and Li^+^ in the presence of BM (−26.6 ± 4.8 pA, *n* = 6, *p* > 0.05) (Figure 6B), indicating Li^+^ directly acts on the post-synaptic hPOA neurons and such responses on hPOA are not due to the activation of GABA_A_, glycine, or ionotropic glutamate receptors.

## 3. Discussion

Results of this study showed that Li^+^ increased GABAergic synaptic activities in hypothalamic preoptic area neurons by action potential independent presynaptic mechanisms as TTX (a voltage-gated Na^+^ channel blocker) did not affect the frequency of spontaneous inhibitory postsynaptic currents (sIPSCs) increased by Li^+^. Besides, Li^+^ perfusion induced inward current for the majority of hPOA neurons. These findings are consistent with previous studies, showing that Li^+^ administration can alter the resting potential [29,30], and enhance GABAergic activities [15].

In the hypothalamic preoptic area, about 70 neuronal populations have been identified with inhibitory neurons being the most abundant [31]. GABAergic and glutamatergic neurotransmissions are the major inputs for neuronal regulation in the hypothalamus [32,33]. Besides, this region has interconnections to various brain areas [11] and receives glycinergic innervation [7,8]. GABA and glycine are major inhibitory transmitters whereas glutamate is the major excitatory transmitter in CNS. Electrophysiological experiments have revealed both excitatory and inhibitory effects of Li^+^ on neuronal excitability [15,29,34].

In electrophysiology, complete or partial replacement of Na^+^ by Li^+^ in the external medium could modulate neuronal properties and a neurotransmitter system. For example, Li^+^ induced depolarization and altered the frequency and shape of the action potential in mitral cells [29]. Similarly, both excitatory and inhibitory synaptic transmission were enhanced by Li^+^ perfusion [15,35,36]. Furthermore, chronic lithium administration resulted in a significant change of the brain neurotransmitter system of selected brain regions [37]. At the neuronal level, Li^+^ exhibited both pre-synaptic and post-synaptic action to modulate neurotransmission mediated by dopamine, GABA, glutamate, and serotonin [14,38]. Li^+^ induced a change in the GABAergic system and GABA receptors have been well documented in various areas of CNS, such as corpus striatum [37], prefrontal cortex [39], hypothalamus [37,40], and dentate gyrus [15].

Findings of the present study suggest that the replacement of Na^+^ by Li^+^ in ACSF could rapidly increase the frequency of synaptic activities without altering their amplitudes. In addition, there was no significant difference between the increased ratio of sIPSCs and mIPSCs frequency induced by Li^+^_,_ indicating that Li^+^ enhanced the action potential-independent presynaptic mechanism mediated GABAergic synaptic events. However, Lee et al. have observed that exposure to Li^+^ (25 mM) could enhance GABAergic synaptic activities by AP-dependent and AP-independent presynaptic mechanisms in hippocampal slices [15]. Our results also revealed that concentration-dependent replacement of Na^+^ by Li^+^ in ACSF increased frequencies of GABAergic mIPSCs without affecting their amplitudes.

In addition to the presynaptic effect, the postsynaptic effect of Li^+^ on neuronal regulation has been reported in various neuronal groups [29,41,42]. Replacement of Na^+^ with Li^+^ can induce depolarization in cortical neurons [29], giant neurons [41], CA1 neurons [43], spinal motoneurons, and olfactory cortex [42]. Similarly, we observed an inward current when LiCl ACSF perfusion was performed. Li^+^ induced non-desensitizing repeatable inward currents. Such responses were preserved in the presence of tetrodotoxin (TTX, a voltage-gated Na^+^ channel blocker) and blocker for amino acid receptors. Previous findings have suggested that Li^+^ can replace Na^+^ ions and pass through neuronal membranes along Na^+^ channels [29,41,44]. In addition, Li^+^ can interact with electrogenic Na^+^/K^+^ pumps as three action sites for Li^+^ interaction has been identified [42]. To induce membrane depolarization, Li^+^ might suppress the activity of an electrogenic Na^+^ pump, reduce the intracellular K^+^ concentration, and increase the release of an excitatory transmitter [42,45,46]. Grafe et.al have demonstrated that Li^+^ can induce a shift of the K^+^ equilibrium potential responsible for membrane depolarization of neurons [42]. In the present study, we observed that Li^+^ directly acted on hPOA neurons independent of voltage-gated Na^+^ channels, GABA_A_, glycine, or ionotropic glutamate receptors. However, we could not elucidate the complete mechanism responsible for the induced inward current.

Neurotransmission disturbances have been reported in several neurological disorders [47,48], and lithium salt is widely used as a therapeutic agent for treating neurological and psychiatric disorders [49,50]. The therapeutic mechanism of Li^+^ involves its neuroprotective effect, neurotropic effect, and neuronal plasticity [13,51]. Lithium exhibits a neuroprotective effect by modulating glutamatergic transmission and inhibiting N-methyl-D-aspartate (NMDA) receptor-mediated calcium influx induced excitotoxicity [52,53]. Besides, Li^+^ can regulate synaptic plasticity by suppressing α-amino-3-hydroxy-5-methyl-4-isoxazolepropionic acid (AMPA) glutamate receptor trafficking [54]. Despite its therapeutic effects on neurological and psychiatric disorders, it can disturb the neuroendocrine axis, mainly in hypothalamic-pituitary-thyroid, hypothalamic-pituitary-gonadal, and hypothalamic-pituitary-adrenal axis in patients under lithium therapy [55,56]. Besides, oral lithium administration can result in Li^+^ accumulation in central neuroendocrine tissues, such as the hypothalamus and pituitary gland [57]. Furthermore, our electrophysiological data showed an increase in GABAergic neurotransmission across the hypothalamic preoptic area upon lithium exposure. This indicates that Li^+^ treatment might directly affect the hypothalamic region of the brain and regulate the release of various neurohormones involved in synchronizing the neuroendocrine axis. However, the mechanism behind the induced inward current on hPOA neurons upon Li^+^ perfusion warrants further investigation.

It has been reported that lithium and anti-epileptic drugs showed side effects on the endocrine system. Alteration in the endocrine system might lead to endocrine complications and related health problems. Theses complications may arise from altered neurotransmission in the hypothalamus. Using the electrophysiology approach, we showed that GABAergic activity was increased across hypothalamic neurons upon Li^+^ exposure, explaining the cause for the disruption in the neuroendocrine axis in patients receiving lithium therapy.

## 4. Materials and Methods

### 4.1. Animals

Electrophysiological experiments were performed using brain slices prepared from immature mice (postnatal day 10 to 25) housed under 12 h light/12 h dark cycle (lights on at 07:00 AM) with free access to food and water. All animal care conditions and experiments were approved by the Institutional Animal Care and Use Committee of Jeonbuk National University with approval number of CBNU-2018-071 (4 September 2018) and CBNU-2019-089 (14 November 2019).

### 4.2. Brain Slice Preparation

Mice were decapitated. Their brains were promptly excised [28] and immersed in ice-cold artificial cerebrospinal fluid (ACSF) with the following compositions (in mM): 126 NaCl, 2.5 KCl, 2.4 CaCl_2_, 1.2 MgCl_2_, 11 D-glucose, 1.4 NaH_2_PO_4_, and 25 NaHCO_3_ (pH 7.4 when bubbled with 95% O_2_ and 5% CO_2_). Coronal brain slices (230 to 300 μm) containing a hypothalamic preoptic area were obtained with a vibratome (VT1200S, Leica biosystems, Wetzlar, Germany) in ice-cold ACSF. These slices were stored in oxygenated ACSF for at least 1 h at room temperature before electrophysiological recording. Various concentrations of LiCl ACSF were prepared by replacing NaCl iso-osmotically with LiCl.

### 4.3. Whole-Cell Patch-Clamp Recording and Data Analysis

Coronal brain slices were transferred to the recording chamber, entirely submerged, and continuously perfused with oxygenated ACSF at a flow rate of 4 to 5 mL/min. Neurons located in the hypothalamic preoptic area were targeted visually with an upright microscope (BX51W1, Olympus, Tokyo, Japan) equipped with Nomarski differential interference contrast optics. Patch pipettes were prepared from thin-wall borosilicate capillary glass (PG52151–4; WPI, Sarasota, FL, USA) using a Flaming/Brown puller (P-97, Sutter Instruments Co., Novato, CA USA). Patch pipettes were filled with an internal solution containing (in mM): 140 KCl, 1 CaCl_2_, 1 MgCl_2_, 10 HEPES, 4 MgATP, and 10 EGTA (pH 7.3 with KOH). Tip resistance of the loaded pipette ranged from 4 to 6 MΩ. Electrode potential was nullified before giga-seal was achieved. Neurons were voltage-clamped at a holding of −60 mV. These signals were sequentially amplified and filtered at 1 kHz with an Axopatch 200B (Molecular Devices, San Jose, CA, USA) and digitized at 1 kHz using a Digidata 1440A interface (Molecular Devices, San Jose, CA, USA). Acquisition and subsequent analysis of acquired data were performed using a Clampex 10.6 software (Molecular Devices) and an Origin 2018 software (OriginLab Corp., Northampton, MA, USA). Neurons displaying a shift in holding current > 5 pA were considered to have responded. All recordings were made at room temperature.

Synaptic events were analyzed and counted using a Mini-Analysis program (version 6.0.7, Synaptosoft, Decatur, GA, USA). Initially, synaptic currents were screened automatically at >10 pA amplitude threshold and then manually accepted or rejected based on the decay time constant (>5 ms) and the rise time constant (<8 ms). The frequency and amplitude of synaptic events in a single neuron were compared using the Kolmogorov-Smirnov test. All values are provided as a mean ± standard error of the mean. Student’s paired *t*-test was used to compare relative mean amplitude and frequency between two groups. Statistical significance was considered when *p*-values were less than 0.05. “*n*” represents the number of neurons recorded.

### 4.4. Chemicals

Chemicals including lithium chloride (LiCl), picrotoxin, strychnine hydrochloride (strychnine), and chemicals for ACSF were purchased from Sigma-Aldrich (St. Louis, MO, USA). Tetrodotoxin citrate (TTX), DL-AP5 (AP5), and CNQX disodium salt (CNQX) were purchased from Tocris Bioscience (Avonmouth, Bristol, UK). Stocks were diluted (usually by 1000 times) to a working concentration in ACSF before bath application.
